# Transcriptional signatures in histologic structures within glioblastoma tumors may predict personalized drug sensitivity and survival

**DOI:** 10.1093/noajnl/vdaa093

**Published:** 2020-08-03

**Authors:** Cymon N Kersch, Cheryl J Claunch, Prakash Ambady, Elmar Bucher, Daniel L Schwartz, Ramon F Barajas, Jeffrey J Iliff, Tyler Risom, Laura Heiser, Leslie L Muldoon, James E Korkola, Joe W Gray, Edward A Neuwelt

**Affiliations:** 1 Department of Neurology, Blood-Brain Barrier Program, Oregon Health and Science University, Portland, Oregon, USA; 2 Department of Biomedical Engineering, OHSU Center for Spatial Systems Biomedicine, Oregon Health and Science University, Portland, Oregon, USA; 3 Knight Cancer Institute, Oregon Health and Science University, Portland, Oregon, USA; 4 Advanced Imaging Research Center, Oregon Health and Science University, Portland, Oregon, USA; 5 Department of Neurology, Layton Aging and Alzheimer’s Disease Center, Oregon Health and Science University, Portland, Oregon, USA; 6 Department of Radiology, Oregon Health and Science University, Portland, Oregon, USA; 7 Department of Neurology and Department of Psychiatry and Behavioral Sciences, University of Washington, Seattle, Washington, USA; 8 Department of Pathology, Stanford University, Stanford, California, USA; 9 Department of Neurosurgery, Oregon Health and Science University, Portland, Oregon, USA; 10 Office of Research and Development, Department of Veterans Affairs Medical Center, Portland, Oregon, USA

**Keywords:** gene signature, glioblastoma, heterogeneity, transcriptomics

## Abstract

**Background:**

Glioblastoma is a rapidly fatal brain cancer that exhibits extensive intra- and intertumoral heterogeneity. Improving survival will require the development of personalized treatment strategies that can stratify tumors into subtypes that differ in therapeutic vulnerability and outcomes. Glioblastoma stratification has been hampered by intratumoral heterogeneity, limiting our ability to compare tumors in a consistent manner. Here, we develop methods that mitigate the impact of intratumoral heterogeneity on transcriptomic-based patient stratification.

**Methods:**

We accessed open-source transcriptional profiles of histological structures from 34 human glioblastomas from the Ivy Glioblastoma Atlas Project. Principal component and correlation network analyses were performed to assess sample inter-relationships. Gene set enrichment analysis was used to identify enriched biological processes and classify glioblastoma subtype. For survival models, Cox proportional hazards regression was utilized. Transcriptional profiles from 156 human glioblastomas were accessed from The Cancer Genome Atlas to externally validate the survival model.

**Results:**

We showed that intratumoral histologic architecture influences tumor classification when assessing established subtyping and prognostic gene signatures, and that indiscriminate sampling can produce misleading results. We identified the cellular tumor as a glioblastoma structure that can be targeted for transcriptional analysis to more accurately stratify patients by subtype and prognosis. Based on expression from cellular tumor, we created an improved risk stratification gene signature.

**Conclusions:**

Our results highlight that biomarker performance for diagnostics, prognostics, and prediction of therapeutic response can be improved by analyzing transcriptional profiles in pure cellular tumor, which is a critical step toward developing personalized treatment for glioblastoma.

**Key Points**

- Variations in histology confound results of established gene signatures.
- Analyzing the cellular tumor improves glioblastoma subtyping into biologically distinct cohorts.
- Patient risk stratification is dramatically improved by analyzing the cellular tumor.

**Importance of the Study**

This paper describes a strategy to improve stratification of patients with glioblastoma by analyzing gene expression patterns in specific histologically defined tumor regions, and draws attention to the critical importance that intratumor histologic variability plays in the development and future clinical use of predictive and prognostic molecular biomarkers.

Glioblastoma is the most common and aggressive malignant primary brain tumor, with a median survival of 18.1 months.^1^ Improving survival has been hindered by the inability to stratify glioblastoma by clinical parameters such as therapeutic sensitivity (chemoradiotherapy [CRT], immunotherapies, and targeted therapies) and rate of disease progression. Identifying patient cohorts with similar glioblastoma tumors would improve preclinical therapeutic development, design of clinical trials, clinical decision-making, and ultimately patient outcomes.

Stratification of glioblastoma is challenging because these tumors display complex multilayered inter- and intratumoral heterogeneity.^2,3^ Current clinical stratification methods include extent of resection, Karnofsky Performance Score (KPS), age, O^6^-methylguanine-DNA methyltransferase (*MGMT*) promoter methylation, and isocitrate dehydrogenase 1 (*IDH1*) mutation, none of which captures the heterogeneous landscape of glioblastoma.^4–9^ Modern “omic” technologies, such as high-throughput genomic, transcriptomic, and proteomic profiling, enable new approaches for tumor subset identification. Omic analyses of glioblastoma samples from The Cancer Genome Atlas (TCGA) defined 4 molecular subtypes: classical, neural, proneural, and mesenchymal.^10^ However, these subtypes and subsequent prognostic gene signatures have not yet found clinical utility.

We explore the hypothesis that glioblastoma intratumoral heterogeneity has impeded the development of robust molecular tools for patient stratification due to sampling regions that differ in histological structure composition. Nearly all omic studies investigating glioblastoma have used samples collected with little regard for histological structure besides necrosis.^11^ This methodology captures nonuniform, varying amounts of histologically diverse tissue architecture composed of cancer cells, stromal cells, vasculature, immune infiltration, and necrosis. Samples that contain a mixture of these elements may obscure detection of key tumorigenic processes enriched or depleted in specific tumor microenvironments. This histologic heterogeneity likely interferes with interpatient comparisons when biopsies are composed of inconsistent tissue architecture.

Herein we utilize an open-source glioblastoma gene expression atlas to characterize transcriptional patterns contributing to intratumoral heterogeneity and demonstrate that (1) histologic structures within a tumor are molecularly distinct and confound results of established gene signatures created from mixed-structure samples and (2) analyzing the dense cellular tumor histological structure improves glioblastoma subtyping into biologically distinct cohorts and patient risk stratification. These advances will guide the development of precision medicine approaches for glioblastoma and enhance prognostics to identify patients with the highest risk of rapid progression.

## Methods

### Data

#### Data sets.

—RNA-sequencing data from newly diagnosed tumors from the Ivy Glioblastoma Atlas Project (Allen Brain Institute) normalized as Fragments Per Kilobase of transcript per Million (FPKM) mapped reads and corresponding clinical data from Swedish IvyGAP Database were used in the present analyses (*n* = 34 subjects) (http://glioblastoma.alleninstitute.org).^12^ Gene expression data (HTSeq-FPKM), methylation data, and corresponding clinical information from the Genomic Data Commons Data Portal for the Glioblastoma Multiforme projects (https://portal.gdc.cancer.gov and https://tcga-data.nci.nih.gov/docs/publications/gbm_2013/) (*n* = 156 newly diagnosed cases).

#### Data preprocessing.

—For the 2 data sets independently, lowly expressed genes with mean values across all samples falling below the lower quartile were filtered out. FPKM values, log2-transformed data, or *z*-score-normalized values were used for analyses herein.

### Variation in Gene Expression Is Primarily Explained by Histologic Structure

#### Principal component analysis and t-distributed stochastic neighbor embedding.

—Principal component analysis (PCA) and t-distributed stochastic neighbor embedding (t-SNE) were performed on the 1000 most variable genes in the IvyGAP data set on log2-transformed and *z*-score-normalized data matrix (FactoMineR, factoextra, and M3C *R* packages).^13^

#### Correlation network analysis.

—A sample-to-sample correlation graph was plotted in BioLayout Express3D. Nodes represent individual samples, and edge length depicts the degree of correlation between samples with Pearson correlation coefficients above *r* = 0.92, for visualization.

#### Gap statistic analysis.

—The gap statistic comparing the total intracluster variation for *k* = 1–10 was determined on the top 1000 most variable genes (factoextra *R* package).^13^ The optimal number of clusters maximizes the gap statistic.

#### *K*-means clustering.

—*K*-means clustering was performed on the 1000 most variable genes in the IvyGAP data set using *k* = 4, the optimal clusters determined by the gap statistic analysis, and visualized by principal components (stats and factoextra *R* package).^13^

#### Dendrogram.

—A distance matrix on the 1000 most variable genes in the IvyGAP data set was computed using Euclidean distance measure and hierarchical cluster analysis by Ward’s method. Dendrogram construction used *k* = 4 groups (stats and factoextra *R* package]).^13^

### Structure-Based Lasso Logistic Regression Classifier

The IvyGAP data set was balanced between structures and evenly split into train and test sets using Stratified K-Folds cross-validator (n_splits = 5). Using train sets, a lasso regularized logistic regression classifier was built to classify glioblastoma multiforme (GBM) structure in independent data sets (penalty = “L1”; solver = “saga”; C = “1/8,” multiclass = “multinomial,” fit intercept = True). The classifier’s cross-validation average accuracy on test sets was 98.45%. The classifier was used to assign the structure classification of all GBM-TCGA samples (class sklearn.linear_model.StratifiedKFolds and .LogisticRegression; Python3.6).^14^

#### Gene set enrichment analysis to assess for enriched biological processes and perform GBM subtype analysis.

—Gene set enrichment analysis (GSEA) was performed using GSEA software with FPKM gene expression data.^15^ Defaults were used for GSEA analysis, including Signal2Noise ranking metrics. Gene sets smaller than 15 genes or greater than 500 genes were excluded, and enrichment *P* values were estimated by 1000 permutations and corrected for multiple testing using the Benjamini–Hochberg method. Analyzed gene sets were from the molecular signature database (MsigDB), Gene Ontology (C5), Hallmark (H), or Positional (C1) collections (www.broadinstitute.org/gsea/msigdb/collections.jsp). For molecular subtypes, single sample GSEA was performed.

GSEA results were visualized using the Enrichment Map plugin for Cytoscape (V2.8, www.cytoscape.org).^16^ For visualization purposes, clusters of functionally related enriched GO terms were manually circled and labeled, and significance thresholds were set to be highly conservative for the LE/IT and HBV/MVP structures (*P* value cutoff 0.005; false discovery rate [FDR] *q*-value cutoff 0.001), conservative for PNZ/PAN (*P* value cutoff 0.005; FDR *q*-value cutoff 0.1), and loose for CT (*P* value cutoff 0.1; FDR *q*-value cutoff 0.4).

#### Survival prediction using an established prognostic gene signature.

—Metagene scores for each sample in the IvyGAP data set (*z*-scored using all samples and within each structure independently) were calculated following methods previously described.^17^ Kaplan–Meier survival analysis was performed using metagene scores to separate all IvyGAP samples into high- (metagene score >0) and low-risk (metagene score <0) groups.

### Cox Proportional Hazards Model for Survival Analysis

#### Univariate analysis.

—Univariate Cox proportional hazards regression was performed using age, gender, *MGMT* methylation status, *IDH1* mutation status, 1p19q deletion status, and KPS score in IvyGAP CT samples (survival *R* package).

#### Multivariate analysis.

—Multivariate Cox proportional hazards regression using *MGMT* methylation status, *IDH1* mutation status, and age was performed. Each gene was assigned a hazard ratio (HR), Wald statistic, and a corresponding *P* value using Cox regression analysis. Genes were selected as candidates significantly associated with survival if the *P* value was < 0.05 (HR ≠ 1). The HR for a given gene >1 was defined as a risk gene, <1 was defined as a protective gene (survival *R* package).

#### Stepwise selection.

—Gene candidates from multivariate analysis were applied to the process of forward stepwise selection. Ten random seeds were generated; for each, IvyGAP CT samples were split into train and test sets using 5-fold cross-validation. Using train sets, HRs, log-rank test scores, and *P* values were computed for the base model, iteratively fit with each candidate gene. An updated model was created adding the candidate gene with the highest log-rank test score to the base model. Iteratively, one of the remaining candidate genes was added that led to the greatest improvement. This process was continued until the concordance = 1 or 10 genes had been added (caret and survival *R* package).

#### Internal validation.

—The model for each train set that underwent stepwise selection was used to predict the HR of the corresponding test set and concordance and log-rank test *P* value was computed. Models were excluded that had concordance <0.5 or log-rank test *P* value >0.05 upon prediction on the test set. The model with concordance nearest the mean (0.75) of all remaining models was selected for subsequent analyses (stats *R* package).

#### Finalized survival model.

—The revised model at the end of the interval validation steps was applied to the entire IvyGAP CT data set and features were excluded that had a Wald statistic *P* value >0.05. The resulting finalized model was trained on IvyGAP CT data and used to predict the HRs for each sample in the IvyGAP CT and the entire IvyGAP data sets (survival and stats *R* package).

#### External validation.

—The finalized survival model was applied to the entire GBM-TCGA and CT-classified data set, as above (stats *R* package).

#### Survival analysis.

—Either an HR of 1 (high-risk: HR > 1; low-risk: HR < 1) or tertiles of HR values were used to classify into 2 (high-risk: HR > quantile (⅔); low-risk: HR < quantile (⅔)) or 3 groups (high-risk: HR > quantile (⅔); medium-risk: quantile (⅓) < HR < quantile (⅔); low-risk: HR < quantile (⅓)). The Kaplan–Meier method was applied to generate survival curves; significance was evaluated using 2-tailed, log-rank tests with *P* values <0.05 considered significant (survival and survminer *R* package).

#### Heatmaps.

—When clustering was performed, transcripts and samples were organized by unsupervised hierarchical clustering using Ward’s method with the Euclidean distance metric. Heatmap visualization and hierarchical clustering were performed on log2-transformed, *z-*score-normalized data (pheatmap *R* package).

## Results

### Histologic Structures in Glioblastoma Are Molecularly Distinct, Contributing to Intratumoral Heterogeneity

**Figure 1.**
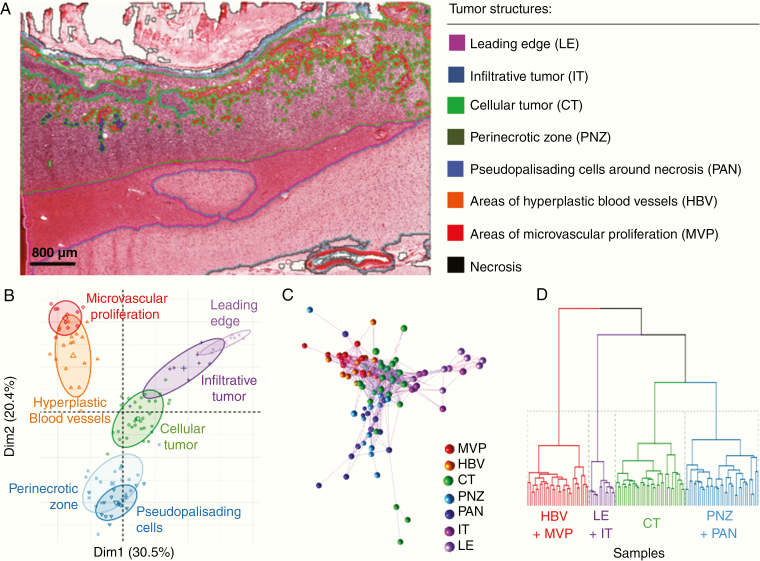
Variation in glioblastoma sample gene expression is primarily explained by histologic structure. (A) Representative image demonstrating the histologic structures that were microdissected, subject to RNAseq and archived in the IvyGAP database by the Allen Brain Institute, scale bar = 800 µm. (B–D) Analysis of the 1000 most variable genes in the IvyGAP data set. (B) Principle component analysis of dimensions 1 (Dim1) and 2 (Dim2) demonstrates that most variation in the data is explained by the histologic structure from which the RNA was extracted. Individual samples (symbol) are colored by the structure they is from; ellipse level = 0.66. (C) Correlation network analysis shows samples (nodes) from a histologic structure cluster. Colors depict the structure samples came from; edge length represents the degree of correlation between samples. (D) Dendrogram of hierarchically clustered (*k* = 4) samples demonstrating structures with the most similarity.

We analyzed RNA-sequencing and corresponding clinical data from the IvyGAP database to compare the transcription profiles of different histological structures.^18^ This database is comprised of 2 companion data sets: (1) RNAseq and In Situ Hybridization data from histologically identified human glioblastoma tumor structures and (2) corresponding clinical information including patient demographics, pathology, and survival. Briefly, predefined histologic structures (infiltrative tumor [IT], leading edge [LE], cellular tumor [CT], perinecrotic zones [PNZ], pseudopalisading cells around necrosis [PAN], areas of hyperplastic blood vessels [HBV], and areas of microvascular proliferation [MVP]) were outlined on H&E stained tumor sections obtained at tumor resection, microdissected on adjacent sections, subject to RNA sequencing, and archived as FPKM mapped reads by the Allen Brain Institute (Figure 1A; Supplementary Figure S1 and Table S1). Necrosis is a characteristic structure in glioblastoma and was a predefined histologic structure in the IvyGAP database; given the lack of cellularity in necrotic tissue, RNAseq was not performed on this structure. The number of different structures sampled varied between patients.

Applying PCA, t-SNE, and correlation network analysis, we analyzed histological structure specific transcriptional profiles from the IvyGAP database.^13,19,20^ The first 2 principal components explained 50.9% of the variance in the 1000 most variable genes and separated samples by structure, but not by other clinical features such as KPS, age, *MGMT* promoter methylation, and *IDH1* mutation (Figure 1B; Supplementary Figure S2A–G). Transcript-to-transcript correlation network analysis and t-SNE corroborated PCA results, confirming that samples within a region were more highly correlated to one another than samples from different regions, even in cases where samples were from the same patient (Figure 1C; Supplementary Figure S2H).^19,20^

Samples from several of the 7 histologically defined structures had overlapping clusters in PCA and network analyses, indicating similarity in their transcription profiles. Applying the gap statistic method, *k*-means clustering, and hierarchical clustering showed that the 7 histologic structures could be collapsed to 4 molecularly distinct structures having high cellularity (CT), tumor invasion (LE/IT), vasculature (HBV/MVP), and necrosis (PAN/PNZ) (Figure 1D; Supplementary Figure S2I and J).

### Distinct Biological Processes Are Enriched in Glioblastoma Structures

**Figure 2.**
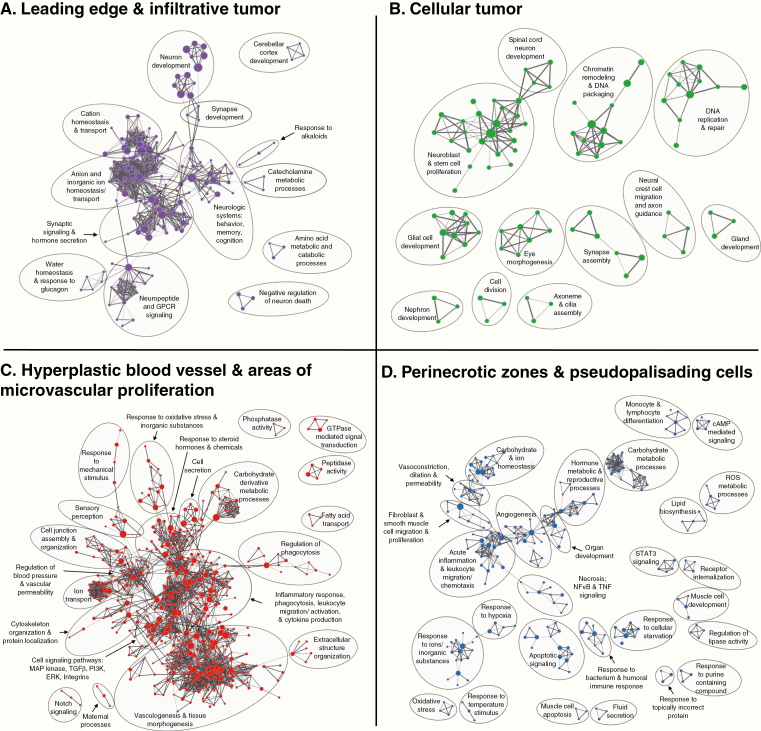
Biological processes enriched in glioblastoma structures. Enrichment map visualizations of GSEA demonstrating gene ontology (GO) biological processes enriched in the (A) LE and IT, (B) CT, (C) HBV and MVP, and (D) PNZ and PAN, relative to the rest of the tumor. Nodes represent GO terms. Functionally related clusters were manually circled and labeled. Node color represents the structure enriched (purple: LE/IT; green: CT; dark orange: HBV/MVP; blue: PNZ/PAN). Node size within each structure quadrant is proportional to the number of genes within each GO term. Edge thickness signifies degree of overlap between GO terms (number of genes shared between 2 gene sets).

We analyzed whole transcriptome measurements of each of the 4 transcriptionally distinct structures applying GSEA of gene ontology (GO) gene sets to identify biological processes enriched in each structure relative to the rest of the tumor (Figure 2).^15,16,21–23^ The LE/IT structure, where the ratio of tumor cells to central nervous system (CNS) cells is low, had enrichment of normal CNS processes such as neuron development, synaptic signaling, and regulation of ion and neurotransmitter homeostasis. Thus, transcriptomic analysis of the bulk tumor edge captures CNS processes, rather than cancer specific biology. This importantly implies that if one inadvertently samples IT or LE tissue for gene expression studies, results will contain a higher contribution of expression data from normal CNS cells rather than the tumor cells, providing a misleading representation of the tumor biology. The vascular architecture (HBV/MVP) was associated with angiogenesis, regulation of blood pressure, vascular permeability, cell junction assembly, and extracellular structure organization. This region also was enriched in immune processes including regulation of phagocytosis, leukocyte migration and activation, and cytokine production, suggesting an inflammatory microenvironment. The PNZ/PAN architecture also was associated with enhanced immune processes, including monocyte and lymphocyte differentiation, and leukocyte migration and chemotaxis. Additionally, the PNZ/PAN region was characterized by biological networks associated with necrosis, cellular starvation, hypoxia, and oxidative stress.

The CT structure has the highest density of neoplastic cells and the transcription profiles of this structure varied between patients. The variation between patients decreased our ability to identify biological processes associated with the overall CT structures. However, there was a trend toward enhancement of traditional cancer processes including DNA replication and repair, chromatin remodeling, and stem cell proliferation. The transcriptional variation observed in this structure, which histologically is a more homogenous distribution of neoplastic cells in all patients, suggests that analysis of gene expression profiles from CT might enable more precise identification of biologically distinct tumors. Thus, we next evaluated the expression patterns of select subtyping and prognostic gene sets in all structures and within the CT.

### Molecular Subtype Classification Depends on Structure, With CT Best Able to Distinguish Subtypes

Glioblastoma molecular subtypes (mesenchymal, classical, neural, and proneural) are not strongly associated with clinical endpoints.^10^ Thus, this gene classifier has not translated clinically. Additionally, several analyses have reported classification of a single tumor into multiple subtypes.^24–26^ We reasoned that these issues might be related to histological heterogeneity within and between tumors, and may be improved by evaluating purely CT tissue.

**Figure 3.**
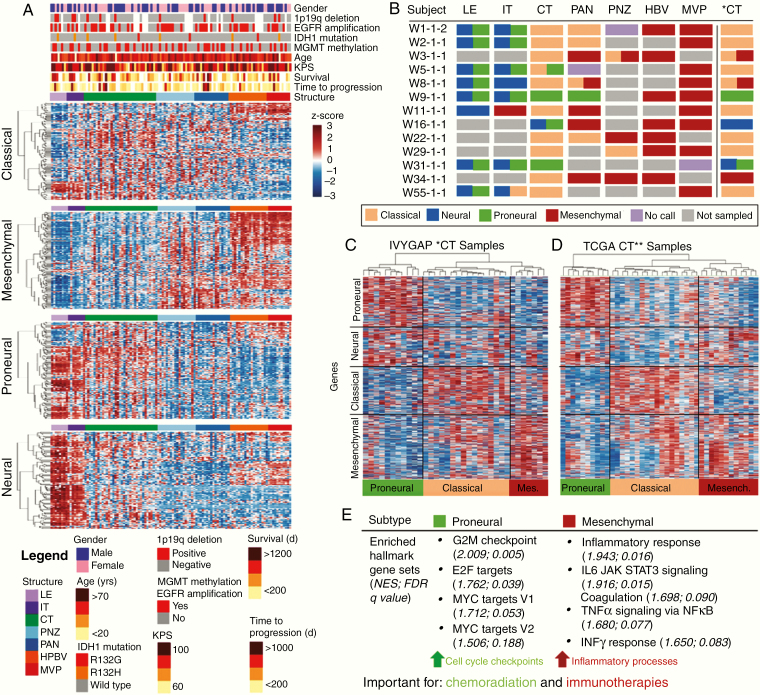
Molecular subtype classification depends on the structure sampled, with CT able to distinguish biologically distinct subtypes. (A) Expression of subtype gene sets (*y*-axis) in IvyGAP samples (*x*-axis) from each region show that sample structure is a main contributor to expression of subtype gene signatures. Genes corresponding to each subtype were organized independently by unsupervised hierarchical clustering. (B) Subtype classification for samples from subjects with data from ≥4 different regions. CT* represents subtype analysis applying data *z*-scored across only CT samples. (C) Unsupervised hierarchical clustering of IvyGAP CT samples (data *z*-scored across CT samples) showing 3 main clusters with signatures of proneural, classical, and mesenchymal subtypes. (D) Unsupervised hierarchical clustering of TCGA CT-classified samples demonstrating 3 main clusters with signatures of proneural, classical, and mesenchymal subtypes. CT** represents TCGA samples predicted to be composed of predominantly CT based on our structure prediction gene signature. (E) Enrichment of hallmark gene sets in proneural and mesenchymal subtypes, characterized based on CT sample analysis. Proneural and mesenchymal tumors have enrichment of cell cycle checkpoints and immune processes, respectively. FDR, false discovery rate; NES, normalized enrichment score.

Our analyses showed that histological architecture significantly influenced subtype classification of samples using previously defined subtype criteria (Figure 3A; Supplementary Figure S3A).^10^ Neural and proneural subtype-defining genes were strongly expressed in LE and IT samples, while mesenchymal subtype genes were highly expressed in HBV and MVP samples. This suggests that a biopsy taken from the tumor edge might be classified as neural or proneural, while a biopsy from the same tumor taken from a highly vascular region might be classified as mesenchymal. Subtyping all samples from each structure, demonstrated that a single patient could be classified as every subtype depending on the structure analyzed. To avoid this problem, we focused on using solely the CT, as this structure showed the most subtype gene expression variability. All 4 subtypes could be distinguished in CT (Figure 3B and C; Supplementary Figure S3B). Our results suggested 3 main subtypes exist: proneural, classical, and mesenchymal (Figure 3C), supporting the idea that the original neural subtype may have been an artifact,^27^ and importantly highlighting that analysis of subtyping gene sets in the CT may permit improved interpatient comparisons.

Using lasso logistic regression on each of the 4 transcriptionally distinct tumor structures in the IvyGAP database we created a novel gene expression classifier to identify expression profiles that distinguish the 4 structures (Supplementary Figure S4A and B).^14^ Applying this new gene classifier to tissue composed of mixed structures identifies the predominant structure in a sample. We applied this structure classifier to glioblastoma samples from TCGA and classified 40 samples as predominantly CT composition (Supplementary Figure S4B).^28^ Clustering these 40 samples revealed proneural, classical, and mesenchymal cohorts, similar in pattern to the IvyGAP CT samples (Figure 3D).

### Molecular Subtype Classification Using CT Distinguishes Tumors With Unique Biology

We performed GSEA on the proneural, classical, and mesenchymal cohorts identified in the CT samples from the IvyGAP database to identify enriched hallmark gene sets in each subtype.^15,29^ The proneural and mesenchymal, but not classical and neural, cohorts had significantly enriched gene sets (Figure 3E; Supplementary Figure S5). Cell cycle checkpoints (G2M and E2F hallmark gene sets) and MYC signaling (MYC targets hallmark gene set) were enriched in proneural tumors, while the mesenchymal tumors were highly inflammatory (enriched inflammatory response, IL6/JAK/STAT3 signaling, coagulation, and interferonγ response gene sets). These patterns were corroborated in the CT-classified TCGA samples (Supplementary Figure S5).

### Survival Prediction Using an Established Prognostic Gene Signature Is Driven by Tumor Structure

**Figure 4.**
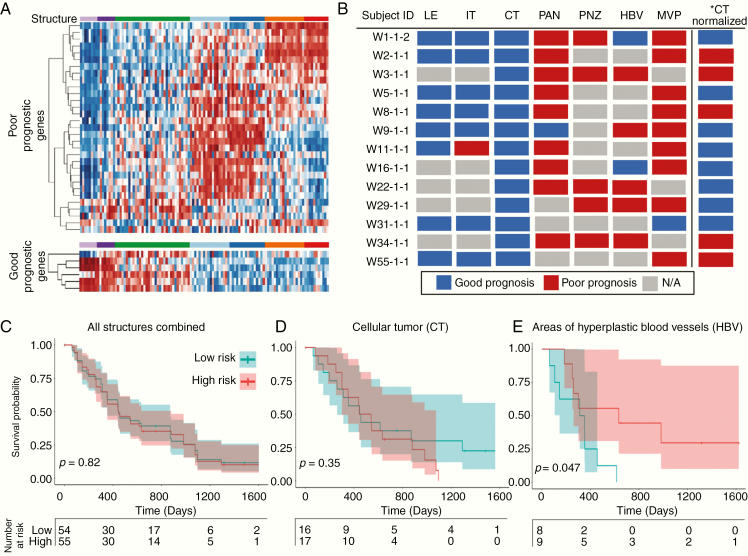
Established prognostic gene signature expression is driven by glioblastoma structure. (A) A survival prediction gene set demonstrates differential expression in tumor structures, with opposite expression in the IT/LE and the PAN/PNZ/HBV/MVP. Transcripts were organized independently by unsupervised hierarchical clustering. (B) Prognostic prediction for samples from subjects with data from ≥4 structures, with prognosis determined by sample metagene score. A single subject can be predicted to have high- or low-risk depending on which structure is analyzed. *CT normalized represents CT samples that underwent z-score normalization utilizing only the CT samples rather than all structure samples. (C) Kaplan–Meier survival analysis of all IvyGAP samples. (D) Kaplan–Meier analysis using a metagene score calculated applying CT samples, demonstrating a trend in survival stratification. (E) Kaplan–Meier analysis using a metagene score calculated applying only HBV samples, significantly and incorrectly stratified long versus short survivors. For survival analyses, metagene scores were used to risk stratify (poor prognosis: metagene score >0; good prognosis: metagene score <0).

We applied an established multigene predictor of glioblastoma outcome to transcriptomic profiles from structurally distinct samples and observed that predicted survival outcome was confounded by structure (Figure 4A; Supplementary Figure S6A).^17^ LE and/or IT samples predicted good prognoses, while PNZ, PAN, HBV, and/or MVP samples predicted poor prognoses, independent of patient origin. Effectively, an individual could be assigned either a good or poor prognosis based on the histological structure analyzed (Figure 4B; Supplementary Figure S6B).^17^ Using the metagene score to separate all IvyGAP samples into high- versus low-risk groups showed no survival difference in Kaplan–Meier analysis, due to a single endpoint being associated with multiple samples that predict opposite outcomes. Independent Kaplan–Meier analyses on samples within each structure demonstrated that CT trended to correctly stratifying patients, while HBV samples significantly (*P* < 0.05) inverted the survival curve, alarmingly predicting a poor prognosis in subjects who had longer survival.

### A Novel Prognostic Gene Signature, Created Utilizing CT Transcriptomics, Identifies Highest Risk Glioblastoma Patients

**Figure 5.**
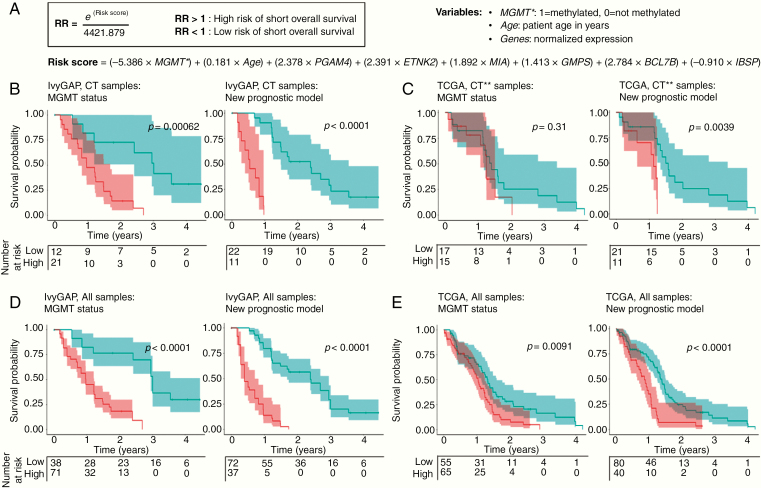
Novel prognostic gene signature created utilizing CT expression data. (A) Risk score and hazard ratio (HR) prediction equation created using a novel prognostic model for glioblastoma. The risk score is the sum of the products of defined weighting factors with the corresponding predictors, *MGMT* promoter methylation status (0: not methylated; 1: methylated), patient age (in years), and normalized expression values of 6 genes. Kaplan–Meier survival analysis of (B) IvyGAP CT samples, (C) CT-predicted TCGA samples, (D) all IvyGAP samples, and (E) all TCGA samples dichotomized into high- and low-risk groups by *MGMT* methylation (left) and predicted HR (right). For *MGMT* methylation, survival was evaluated by separating samples into methylated (low-risk) or unmethylated (high-risk) groups. Tertiles of HR values were used to risk stratify (high-risk: HR > quantile (⅔); low-risk: HR < quantile (⅔)). Shading on survival lines correspond to 95% confidence intervals. *MGMT promoter methylation status. **Samples predicted to be predominantly CT.

We next asked whether patients could be better stratified according to outcome using gene expression profiles measured for the cancer cell rich CT structure. Stepwise multivariate Cox proportional hazards regression on IvyGAP CT samples was used to create a novel glioblastoma prognostic model and risk score equation (Figure 5A; Supplementary Figure S7 and Table S2). We included known prognostic factors including age, *MGMT* status, and *IDH1* mutation in the model. The final risk score calculation included MGMT status, age, and expression of 6 genes: *PGAM4*, *ETNK2*, *MIA*, *GMPS*, *BCL7B*, and *IBSP*.

We assessed HRs in samples from IvyGAP and validated these using the CT samples in the TCGA data set in order to determine whether our prognostic signature improved survival prediction over *MGMT* methylation status alone. Stratification of patients into moderate versus highest risk groups was statistically significant and better than *MGMT* status alone (Figure 5B and C). When applied to all samples from IvyGAP and TCGA, the model correctly stratified patients, again improving stratification over *MGMT* status alone (Figure 5D and E; Supplementary Figure S8). Thus, our survival prediction model, created based on CT gene expression, can be applied to samples containing either pure CT or mixed structures. The model also effectively identified medium- and low-risk groups and when excluding IDH-mutant (Supplementary Figures S8 and S9).

We asked whether genes associated with high-risk had enriched biological patterns that highlight key tumorigenic processes. To test this, we ranked the entire transcriptome in order of the Wald statistic calculated during multivariate Cox regression analysis. Hallmark pathways, including oxidative phosphorylation, MYC targets, MTORC1 signaling, Glycolysis and DNA repair, and genes at chromosomal locations Chr13q12, ChrXp11, Chr16p12, Chr3q22, and Chr3q25 were associated with high-risk status (Supplementary Figure S10).

## Discussion

Improving outcomes for glioblastoma is hindered by our inability to stratify patients into cohorts that have biologically distinct tumors requiring different clinical care. Patient-to-patient tumor comparisons are problematic in glioblastoma due to intratumoral heterogeneity. We demonstrated that histologic structures account for part of this heterogeneity, and propose that assessing gene expression in CT will improve intertumoral comparisons. Our results highlight that using mixed-structure samples or samples rich in non-CT regions to determine glioblastoma subtype could produce invalid results, while classifying subtypes using CT identifies distinct cohorts with unique biology. Additionally, utilizing exclusively CT, we created a prognostic model to identify the highest risk patients. The biological patterns uncovered in the subtypes and risk-stratified groups have important implications for guiding precision medicine and steering future studies investigating malignant pathways in glioblastoma.

The enriched biological processes we identified in glioblastoma subtypes have potential future implications that could help to guide therapeutic intervention. Proneural tumors showed enrichment of genes expressed during cell cycle checkpoints, stages of cell replication when DNA integrity is assessed. Current standard-of-care treatment for glioblastoma is CRT, which functions through eliciting DNA damage.^1^ Having elevated expression of cell cycle checkpoints makes it plausible that proneural tumors have different sensitivity to CRT than other subtypes. Accordingly, purely proneural tumors have been reported to have longer survival than other glioblastomas, while mesenchymal differentiation has been associated with therapeutic resistance and decreased survival.^24,30,31^ In contrast, mesenchymal tumors had enriched immune processes, consistent with previous reports showing that mesenchymal glioblastoma has elevated immune activation and leukocyte infiltration.^32–34^ Highly immunogenic tumors have been found to be more responsive to immunotherapy than tumors with a weak endogenous immune response.^35^ Thus, mesenchymal glioblastoma may be exceptional candidates for single-agent immunotherapy, whereas proneural tumors may require addition of an immune stimulating agent.^34,36,37^ Future studies should investigate the influence of pretreatment CT-characterized subtype, levels of cell cycle checkpoint transcripts and immune phenotype on glioblastoma therapeutic sensitivity.

Our analyses of the established prognostic gene signature suggest that structure composition contributes to its prognostic value. Colman et al. noted that worse prognosis in their gene set was associated with a mesenchymal–angiogenic phenotype.^17^ This observation is supported by our findings that vascular tissue and mesenchymal phenotype have poor prognostic signatures. Multiple studies corroborate that more angiogenic and necrotic glioblastomas may be more aggressive.^30,38,39^ It is plausible that the relative amount of these regions within glioblastoma may be prognostic themselves, perhaps secondary to rapid tumor proliferation.

Using CT to create a novel prognostic gene signature allowed us to identify the highest risk patients and probe their underlying biology. Among the pathways we identified in the high-risk genes, MYC targets are attractive because MYC has multiple protumorigenic functions in glioblastoma.^40,41^ There are currently no clinically viable MYC inhibitors.^42^ Developing these inhibitors may have utility in treating the most aggressive glioblastomas. Additionally, multiple metabolic pathways were associated with high-risk of rapid progression (Supplementary Figure S9A). Previous work demonstrated a link between metabolic signatures and glioblastoma subtypes and outcomes.^43^ Collectively, this highlights the importance of glioblastoma subtypes as possibly harboring distinct biology, bioenergetics, proliferative capacity, immune interaction, and disease progression, all of which are appreciable when accounting for structural variability in tumor analysis. Expression of genes from specific chromosomal locations were also enriched in the high-risk group. As *MGMT* promoter methylation is correlated with survival outcomes in glioblastoma,^5^ it is plausible that unappreciated more widespread epigenetic modifications also exist and drive tumor progression.^44^ Studies have investigated global methylation in glioblastoma^45^; we propose that analyzing these patterns in CT may expose novel drivers of glioblastoma malignancy and provide modifiable therapeutic targets.

As we have shown, CT-based transcriptomics permit interpatient comparisons. This method can be applied to developing predictive gene signatures for clinical utility in glioblastoma, and potentially other heterogeneous cancers. The next steps include (1) creating predictive signatures for tumor sensitivity and response to treatments and (2) identifying methods to collect CT without microdissection. Using CT for clinical purposes will likely be hindered by the labor-intensive microdissection. Using macrodissection or image-guided biopsy may overcome this problem. Diffusion weighted MRI and amino acid positron tomography are already being integrated into the operating room and can localize glioblastoma regions with elevated tumor cellularity and mitotic indices prior to resection, allowing for selective CT localization and biopsy.^46–48^

We have shown that analysis of transcriptomics in CT can stratify patients into distinct cohorts, and that using mixed-structure samples can give misleading information. Ultimately, we believe the present study is a critical step in generating a novel set of transcriptomic-based clinical tools utilized to plan and execute optimal care for glioblastoma patients.

## Supplementary Material

vdaa093_suppl_Supplementary_Figure_S1

vdaa093_suppl_Supplementary_Figure_S2

vdaa093_suppl_Supplementary_Figure_S3

vdaa093_suppl_Supplementary_Figure_S4

vdaa093_suppl_Supplementary_Figure_S5

vdaa093_suppl_Supplementary_Figure_S6

vdaa093_suppl_Supplementary_Figure_S7

vdaa093_suppl_Supplementary_Figure_S8

vdaa093_suppl_Supplementary_Figure_S9

vdaa093_suppl_Supplementary_Figure_S10

vdaa093_suppl_Supplementary_Material

## Data Availability

All computer code used in this work is free and open-source software available at https://github.com/gbm-dx. IvyGAP data were acquired from http://glioblastoma.alleninstitute.org. TCGA data were acquired from https://portal.gdc.cancer.gov and https://tcga-data.nci.nih.gov/docs/publications/gbm_2013/.

## Notes

**Conflict of interest statement**. J.W.G. is employed by Oregon Health & Science University and at Emeritus Professor of Laboratory Medicine at the University of California San Francisco. Licensed technologies to Abbott Diagnostics and Danaher (formerly Cepheid). Having ownership positions in Convergent Genomics, KromaTid and PDX Pharmaceuticals. Served as an advisory capacity and scientific collaborator to Thermo Fisher Scientific (formerly FEI), Miltenyi, Zeiss, KromaTid, and Micron technologies. Served as a paid consultant to New Leaf Ventures. Served in an advisory capacity to the APOBEC Cancer Program, University of Minnesota; Big Data to Knowledge Center for Causal Discovery (BD2K CCD), University of Pittsburgh Cancer Institute & Carnegie Mellon University (Joint); Comprehensive Cancer Center, University of New Mexico (UNM); and the MJ Murdock Charitable Trust. J.W.G. own stock in Abbott Diagnostics, AbbVie, Alphabet, Amazon, Amgen, Ani Pharma, Apple Corporation, Berkshire Hathaway, Cameco, Chevron, Cisco, Clorox, Colgate-Palmolive, Conoco Phillips, Crown Castle, Devon Energy, Disney, Exxon Mobile, Facebook, General Electric, Gilead, Intel, Linde, Microsoft, Nasdaq, Nike, Nvidia, PepsiCo, Philips, Procter & Gamble, Taiwan Semicnductor and Zimmer Biomet. All other authors declare there are no conflict of interest.

## Notes

**Authorship Statement**. Hypothesis generation and project design: C.N.K., C.J.C., P.A., E.B., J.J.I., D.L.S., R.F.B., J.E.K., J.W.G., and E.A.N.; database mining: C.N.K., C.J.C., and E.B.; data analysis and interpretation: C.N.K., C.J.C., P.A., E.B., D.L.S., T.R., R.F.B., L.H., J.E.K., J.W.G., and E.A.N.; manuscript writing: C.N.K., C.J.C., P.A., E.B., L.L.M., J.W.G., and E.A.N.; critical revision of the article for intellectual content: C.N.K., C.J.C., P.A., E.B., D.L.S., T.R., R.F.B., J.J.I., L.H., L.L.M., J.E.K., J.W.G., and E.A.N.
