# Supplementary figures and images for: Transcriptional signatures in histologic structures within glioblastoma tumors may predict personalized drug sensitivity and survival
Source: Neurooncol Adv. 2020 Aug 3;2(1):vdaa093. doi: 10.1093/noajnl/vdaa093 (PMC7462280; doi:10.1093/noajnl/vdaa093)

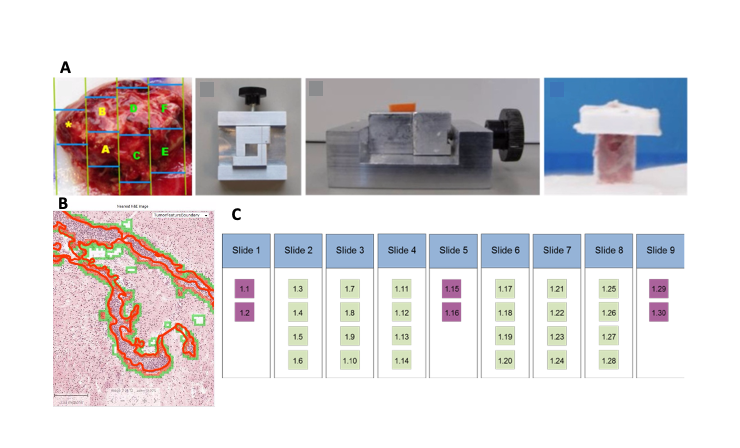

Supplement: vdaa093_suppl_Supplementary_Figure_S1 [file vdaa093_suppl_supplementary_figure_s1.png]

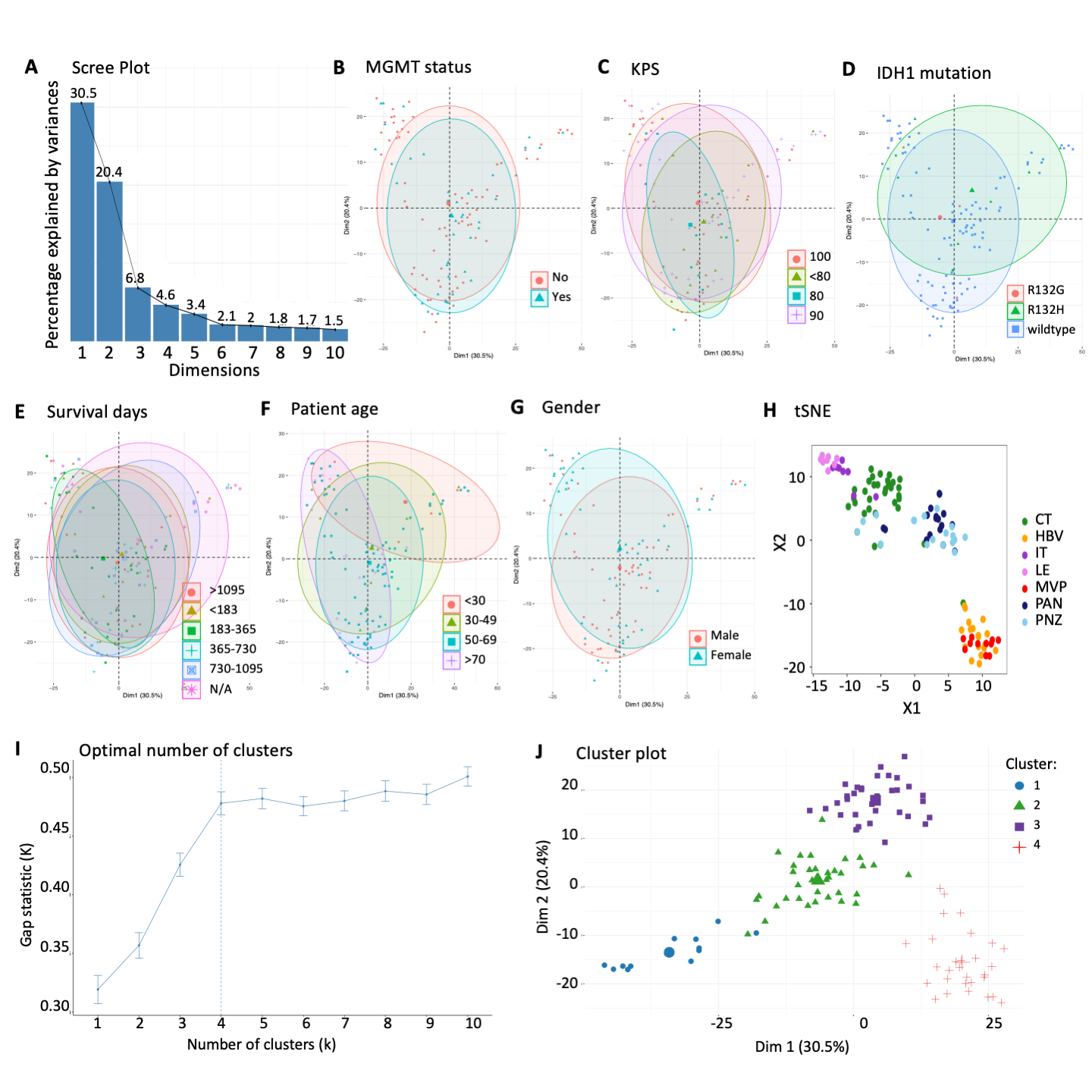

Supplement: vdaa093_suppl_Supplementary_Figure_S2 [file vdaa093_suppl_supplementary_figure_s2.png]

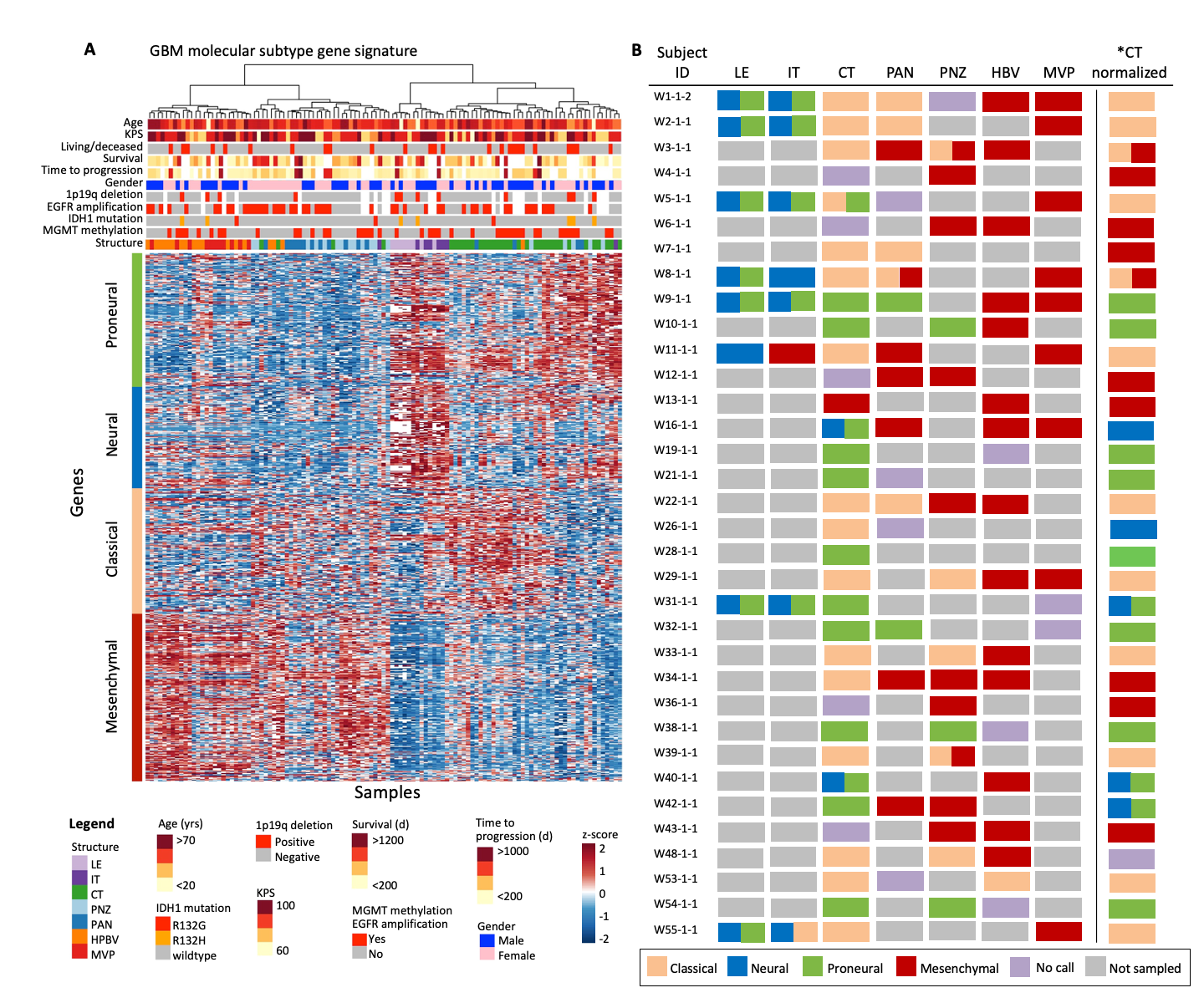

Supplement: vdaa093_suppl_Supplementary_Figure_S3 [file vdaa093_suppl_supplementary_figure_s3.png]

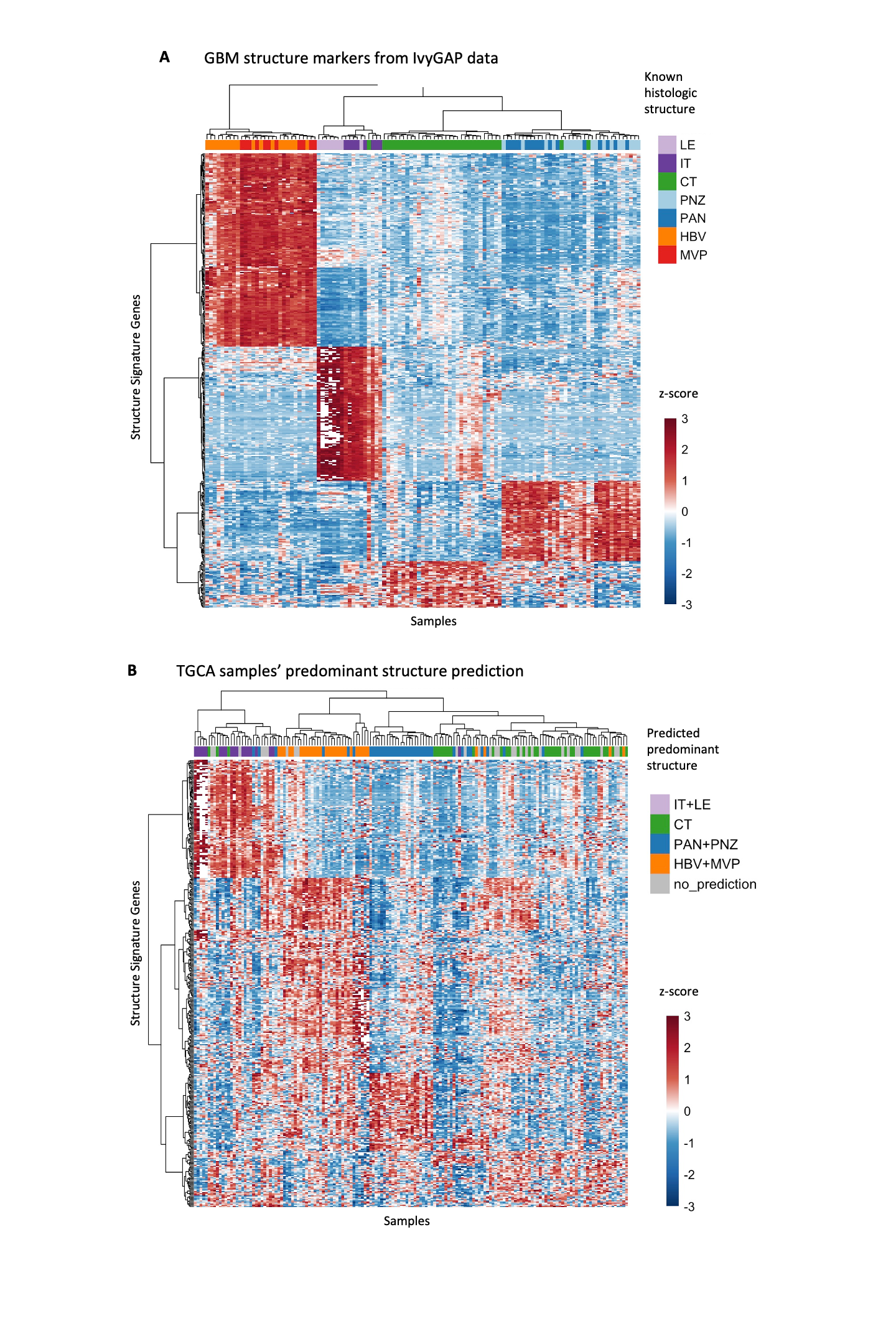

Supplement: vdaa093_suppl_Supplementary_Figure_S4 [file vdaa093_suppl_supplementary_figure_s4.png]

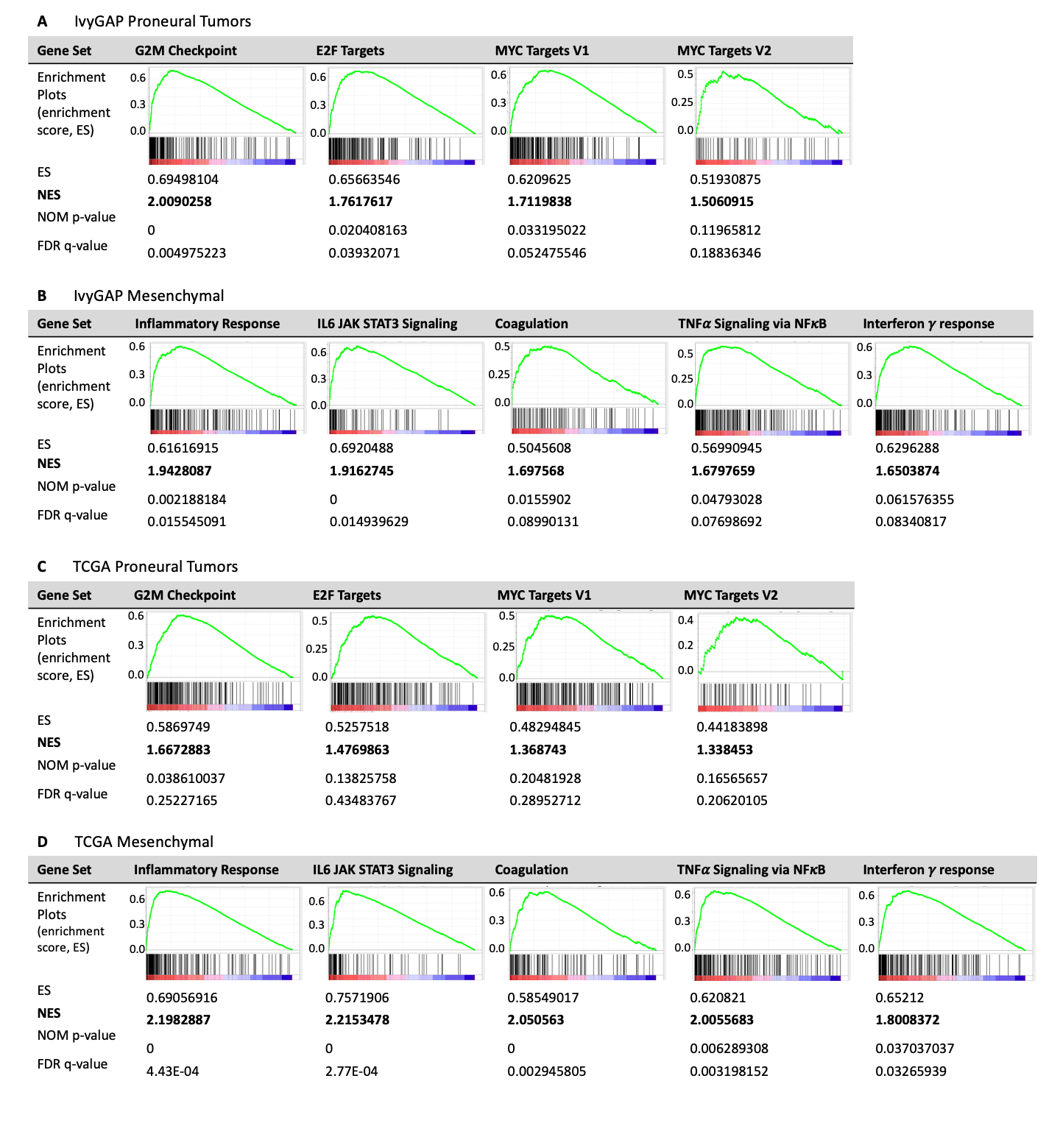

Supplement: vdaa093_suppl_Supplementary_Figure_S5 [file vdaa093_suppl_supplementary_figure_s5.png]

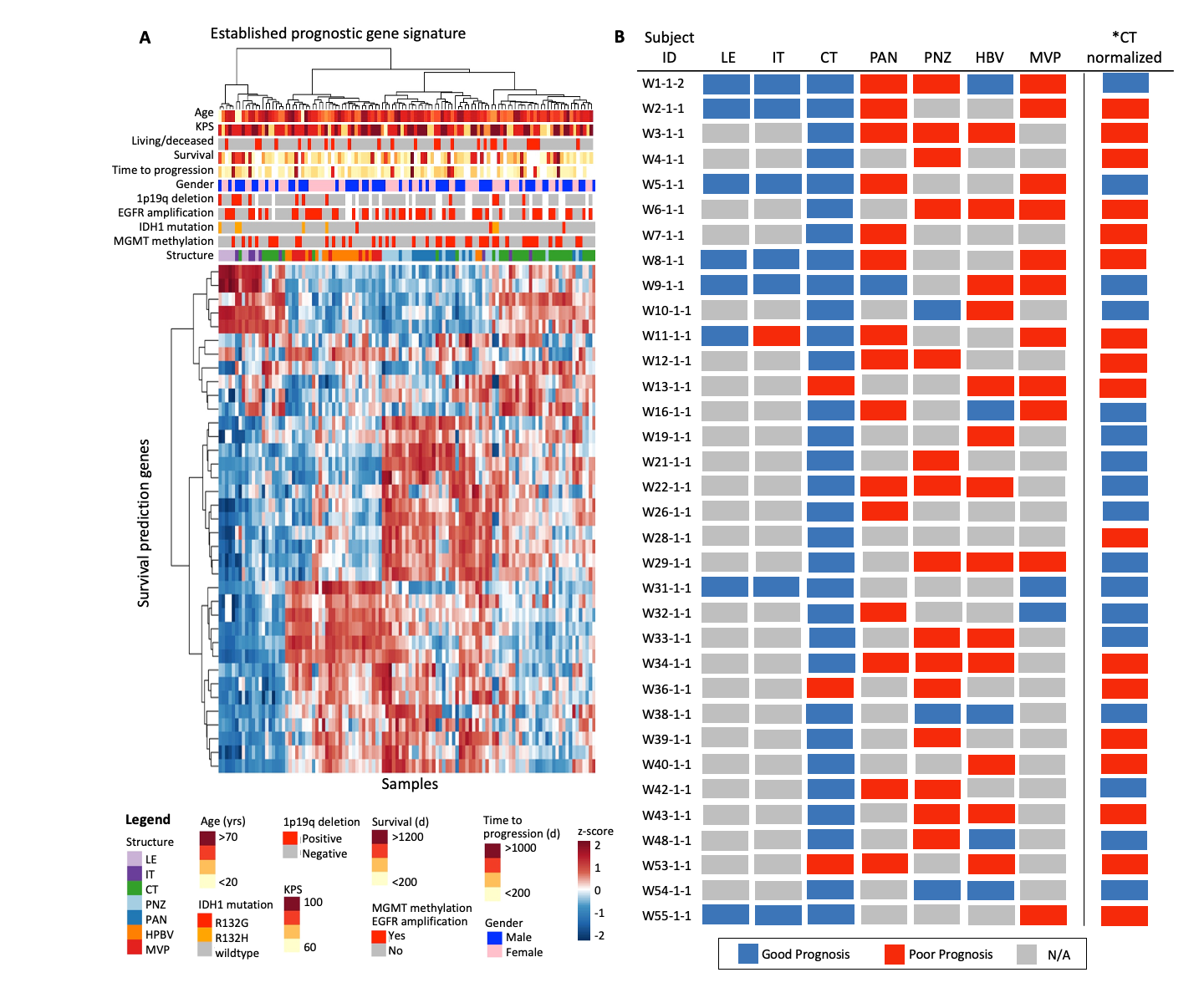

Supplement: vdaa093_suppl_Supplementary_Figure_S6 [file vdaa093_suppl_supplementary_figure_s6.png]

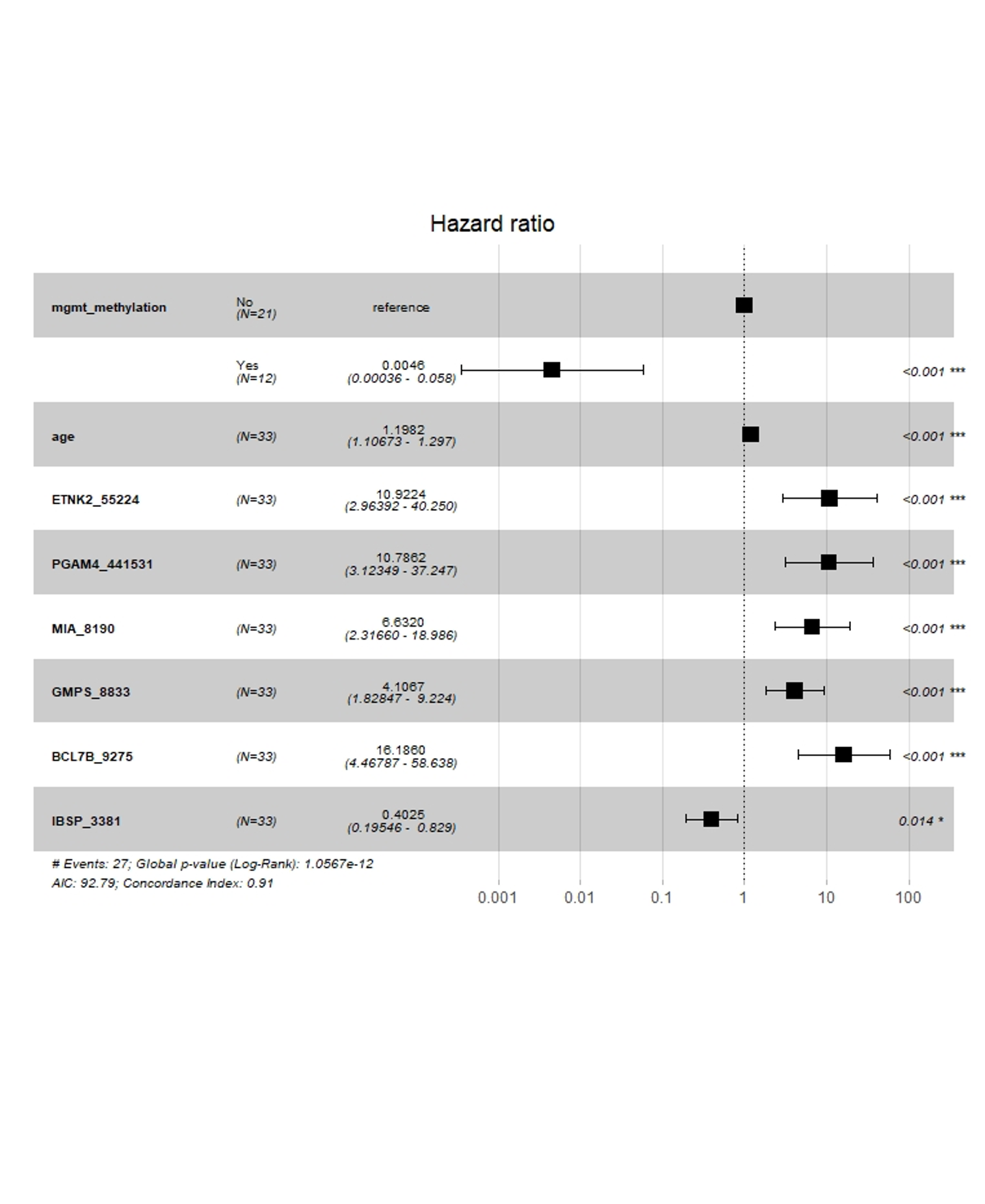

Supplement: vdaa093_suppl_Supplementary_Figure_S7 [file vdaa093_suppl_supplementary_figure_s7.png]

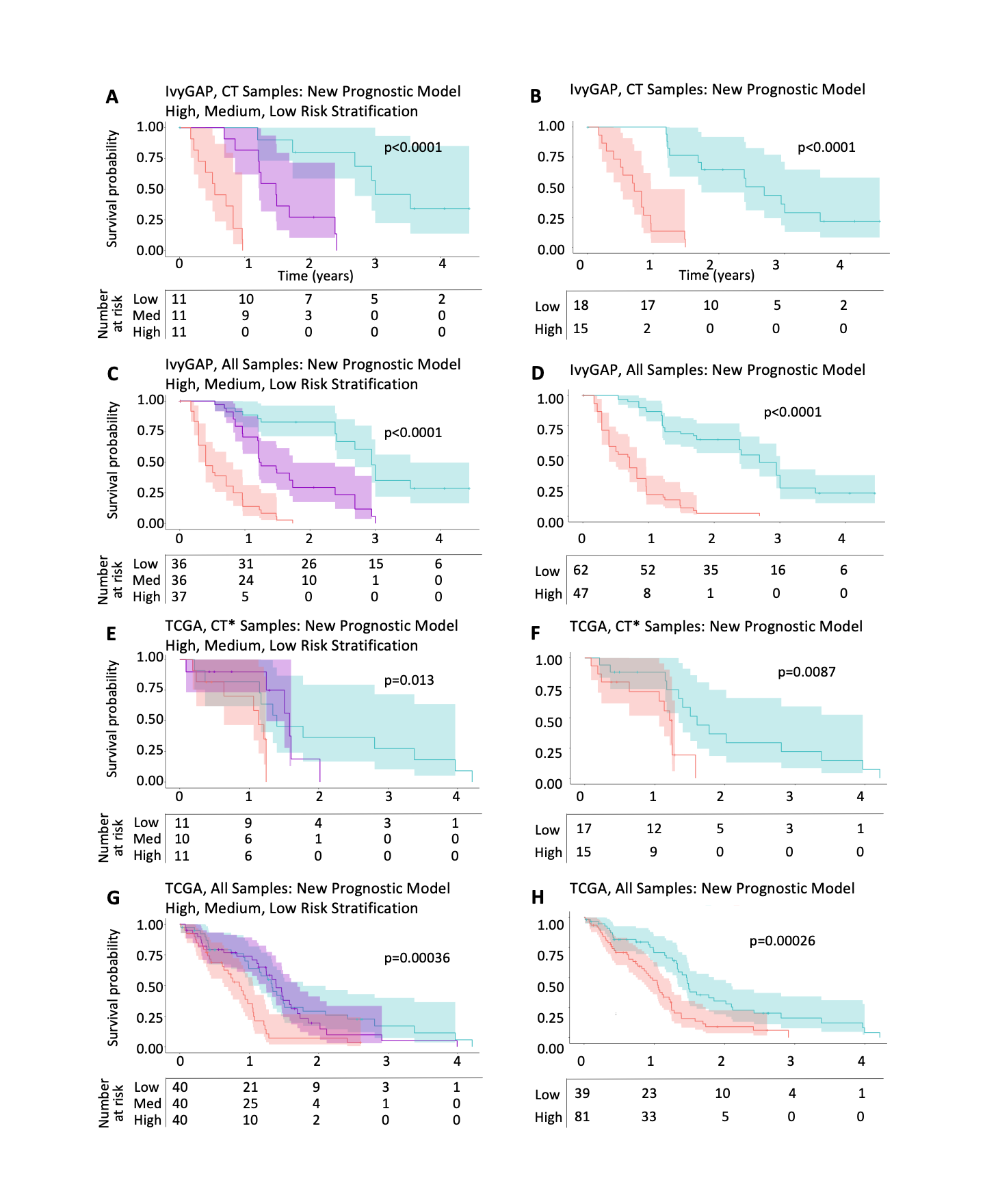

Supplement: vdaa093_suppl_Supplementary_Figure_S8 [file vdaa093_suppl_supplementary_figure_s8.png]

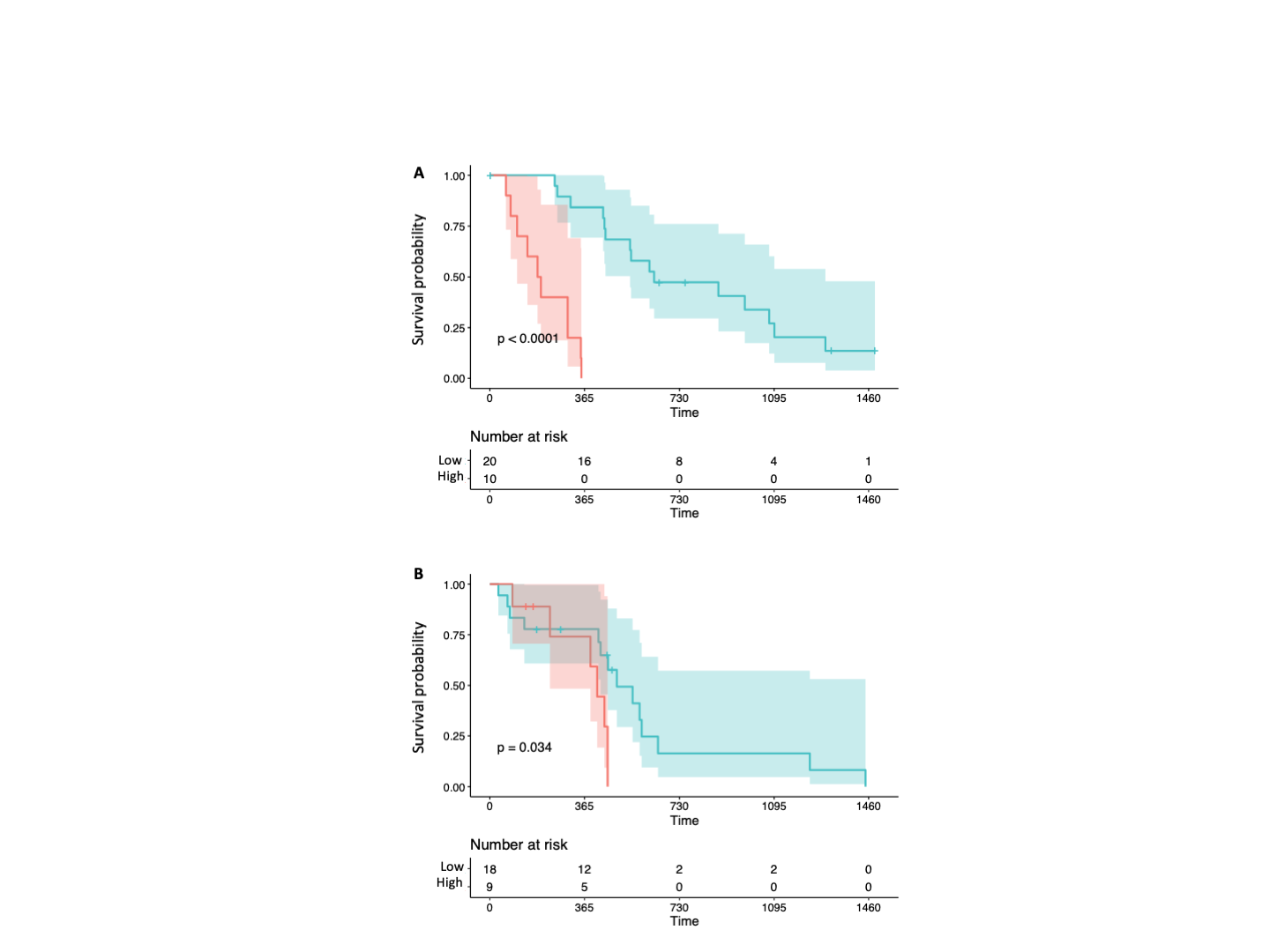

Supplement: vdaa093_suppl_Supplementary_Figure_S9 [file vdaa093_suppl_supplementary_figure_s9.png]

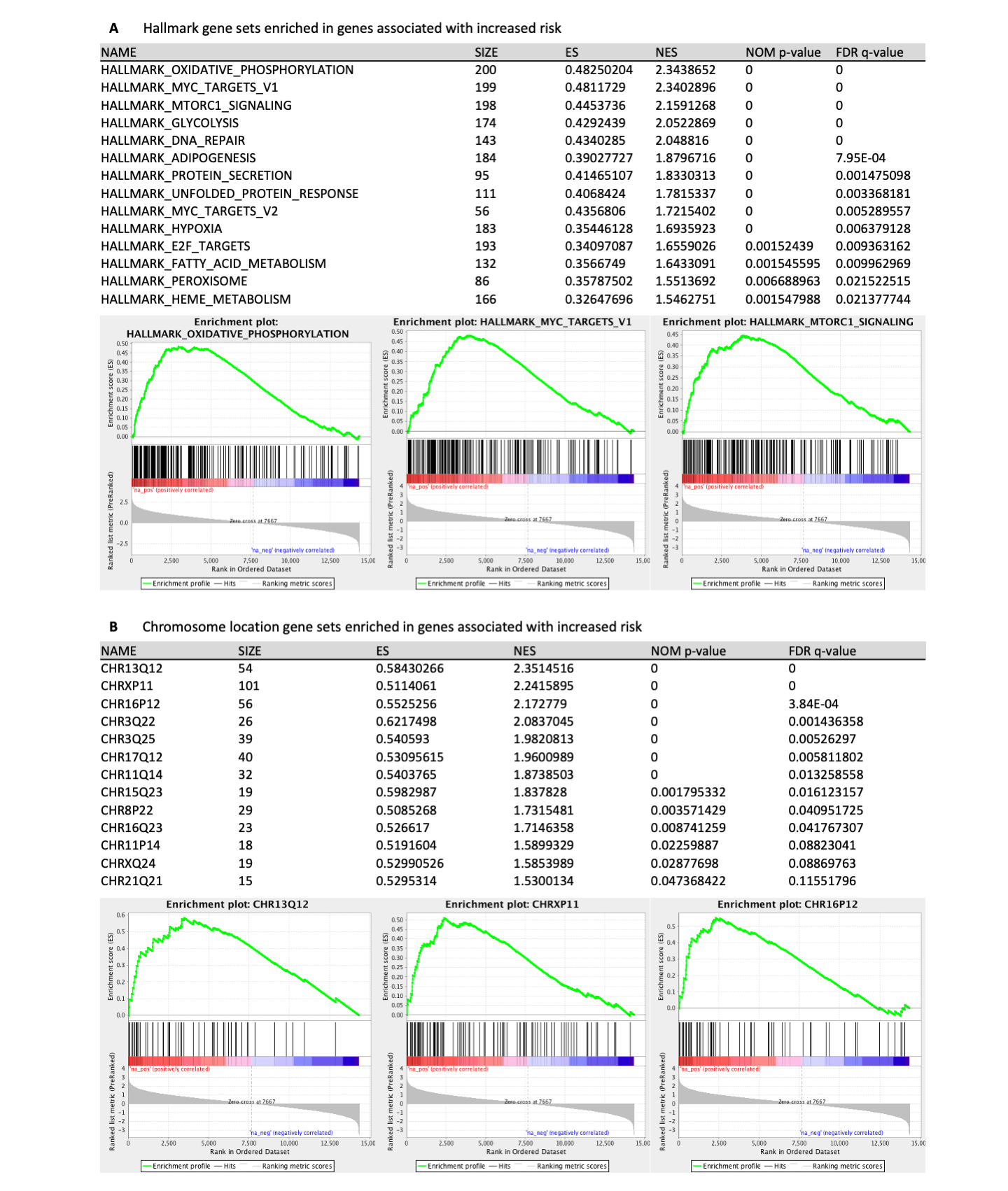

Supplement: vdaa093_suppl_Supplementary_Figure_S10 [file vdaa093_suppl_supplementary_figure_s10.png]
