# Supplementary material for: Transcriptional signatures in histologic structures within glioblastoma tumors may predict personalized drug sensitivity and survival
Source: Neurooncol Adv. 2020 Aug 3;2(1):vdaa093. doi: 10.1093/noajnl/vdaa093 (PMC7462280; doi:10.1093/noajnl/vdaa093)
Supplement: vdaa093_suppl_Supplementary_Material [file vdaa093_suppl_supplementary_material.docx]

**Supplementary Files**

**Supplementary Figure Legends and Tables:**

**Fig. S1.** **Tissue collection and processing.** (**A**) Tissue collection, sub-sectioning, and freezing done by the Allen Brain Institute (ABI). (**B**) Example of histologic structure identification done by the ABI. (**C**) Slide layout for serial sections for structure alignment, completed by the ABI. Images: ABI, technical white paper.

**Fig. S2. Additional PCA and clustering analyses.** (**A**) Scree plot showing percent of variance described by each principle component (dimension) in analysis of the top 1000 most variable transcripts. PCA labeling clinical patient stratifiers (**B**) *MGMT* methylation status, (**C**) Karnofsky Performance Score (KPS), (**D**) *IDH1* mutation status, (**E**) overall survival days, (**F**) patient age at time of diagnosis, and (**G**) gender of the samples (each symbol represents and individual sample). No alternative labeling explains variance in the data set as well or better than histologic structure seen in figure 1. (H) tSNE plot labeling samples by histological structure. (**I)** Gap statistic method identifying optimal number of clusters for k=1-10. (**J**) K-means clustering using k=4 and visualization of clusters using PCA.

R1.5

**Fig. S3. IvyGAP molecular subtyping.** (**A**) Expression of subtype gene set (y-axis) in the IvyGAP samples from each region (x-axis) showing sample structure is a main contributor to expression of subtype gene signatures. Samples were organized by unsupervised hierarchical clustering using Ward’s method and the Euclidean distance metric. (**B**) Subtype classification calls for structures from all samples. CT* represents subtype calls using CT z-scored data across only the CT samples.

**Fig. S4. Structure-based gene signature.** (**A**) Heatmap displaying z-score normalized expression of the structure-based genes signature, which was created by logistic regression modeling on the IvyGAP data with known tumor structures. (**B**) Heatmap of the structure-based signature genes in the TCGA glioblastoma data. The predominant structure was predicted by applying the model learned by logistic regression from the IVGAP data to the TCGA glioblastoma data. (**A**,**B**) Samples and genes were organized by unsupervised hierarchical clustering, which results in a nearly perfect separation of the structure (A). Genes are on the y-axis, samples on the x-axis.

**Fig. S5. GSEA hallmark gene set enrichment results from CT stratified molecular subtypes.** Enrichment plots of top enriched hallmark gene sets in IvyGAP CT samples from (**A**) Proneural versus REST (all samples not classified as proneural), and (**B**) Mesenchymal versus REST (all samples not classified as mesenchymal) analyses. The top enriched hallmark gene sets in IvyGAP CT samples were also enriched in TCGA CT-predicted samples as shown by enrichment plots of (**C**) Proneural versus REST, and (**D**) Mesenchymal versus REST analyses. No results were statistically significant in Classical or Neural versus REST in both IvyGAP and TCGA analyses. Molecular subtyping was determined after z-score normalizing within only the IvyGAP CT and TCGA CT-predicted samples. ES: Enrichment score; NES: Normalized enrichment score; NOM: Nominal; FDR: False discovery rate.

**Fig. S6. Analysis of established prognostic gene signature expression with all samples.** (**A**) A survival prediction gene set, composed of genes associated with poor and good prognosis, shows differential expression based on tumor structure, with opposite expression in IT/LE compared to PAN/PNZ/HBV/MVP. Samples and genes were both organized by unsupervised hierarchical clustering. (**B**) Survival prediction for each sample, with prognosis determined based on sample metagene score (poor prognosis: metagene score > 0; good prognosis: metagene score < 0).

**Fig. S7. Forest plot of new prognostic marker gene signature predictors.** The mid-point of each black box represents the hazard ratio of each predictor with line width representing the 95% confidence interval.

**Fig. S8. Survival analysis using the new survival prediction gene signature**. Kaplan-Meier survival analysis of (**A**,**B**) IvyGAP CT samples, (**C**,**D**) all IvyGAP samples, (**E,F**) CT-predicted TCGA samples, and (**G,H**) all TCGA samples. Based on predicted HR, samples were separated into high-, medium-, and low-risk groups (**A**,**C**,**E**,**G;** high-risk: HR > quantile(^2^/_3_); medium-risk: quantile(^1^/_3_) < HR < quantile(^2^/_3_); low-risk: HR < quantile(^1^/_3_)), or high and low-risk (**B**,**D**,**F**,**H**; high-risk: HR > 1; low-risk: HR < 1). Differences between survival curves was evaluated using the log-rank test. All tests were two-tailed, and p-values less than 0.05 were considered to be significant. Shading on survival lines correspond to 95% confidence intervals. *Predicted to be predominantly CT as classified using the structure-based lasso logistic regression classifier.

**Fig. S9. Survival analysis using the new survival prediction gene signature in only IDH wildtype tumors.** Kaplan-Meier survival analysis of only IDH-wildtype tumors in (A) IvyGAP CT samples and (B) CT-predicted TCGA samples. Tertiles of HR values were used to risk stratify (high-risk: HR > quantile (^2^/_3_); low-risk: HR < quantile (^2^/_3_)). Shading on survival lines correspond to 95% confidence intervals.

**Fig. S10. Enriched gene sets in IvyGAP CT genes associated with increased risk.** (**A**) Hallmark and (**B**) chromosome location gene sets enriched in genes associated with high-risk of short overall survival with enrichment plots of the top 3 gene sets for each. ES: Enrichment score; NES: Normalized enrichment score; NOM: Nominal; FDR: False discovery rate.

**Table S1.** Tumor structure definitions adapted from IvyGAP Technical White Paper Figures 2-9.

| Structure | Example Images | Definition |
| --- | --- | --- |
| Leading Edge (LE) | 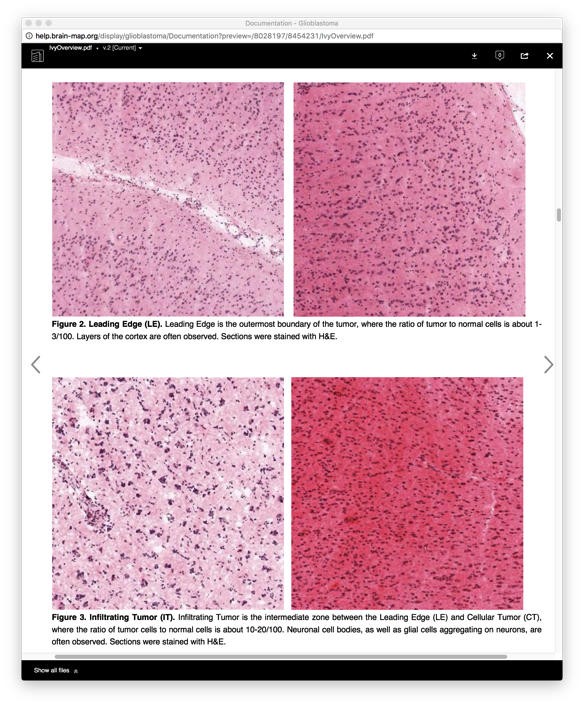 | “Leading Edge is the outermost boundary of the tumor, where the ratio of tumor to normal cells is about 1-3/100. Layers of the cortex are often observed.” |
| Infiltrating Tumor (IT) | 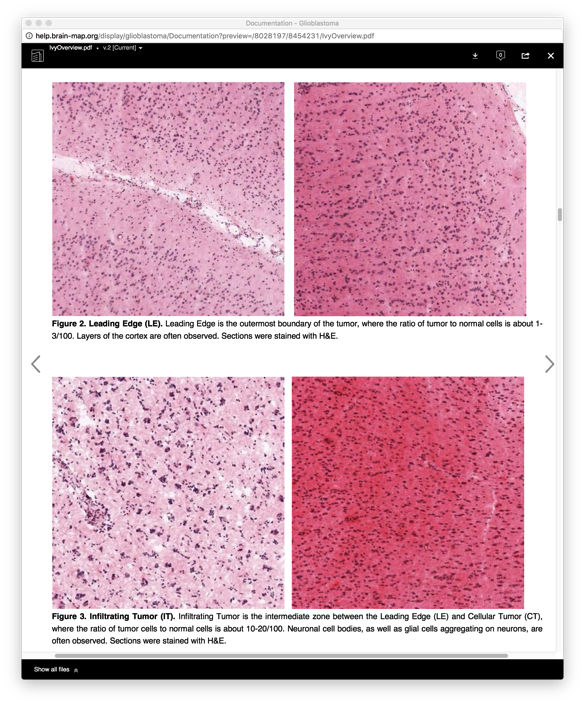 | “Infiltrating tumor is the intermediate zone between the Leading Edge (LE) and Cellular Tumor (CT), where the ratio of tumor cells is about 10-20/100. Neuronal cell bodies as well as glial cell aggregating on neurons, are often observed.” |
| 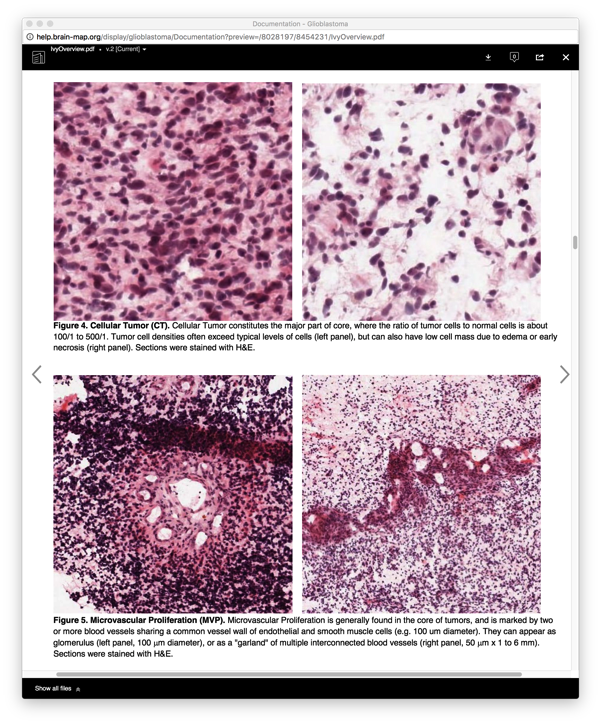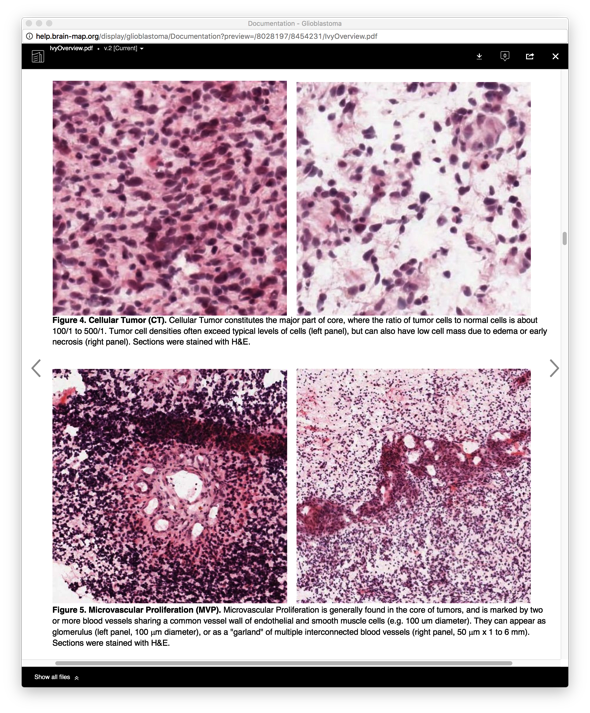Cellular Tumor (CT) |  | “Cellular tumor constitutes the major part of core, where the ratio of tumor cells to normal cells is about 100/1 to 500/1. Tumor densities often exceed typical levels of cells (left panel), but can also have low cell mass due to edema or early necrosis (right panel).” |
| Microvascular Proliferation (MVP) |  | “…generally found in the core of tumors, and is marked by two or more blood vessels sharing a common vessel wall of endothelial and smooth muscle cells (e.g. 100 μm diameter). They can appear as glomerulus (left panel, 100 μm diameter), or as a “garland” of multiple interconnected blood vessels (right panel, 50 μm diameter x 1-6 mm).” |
| 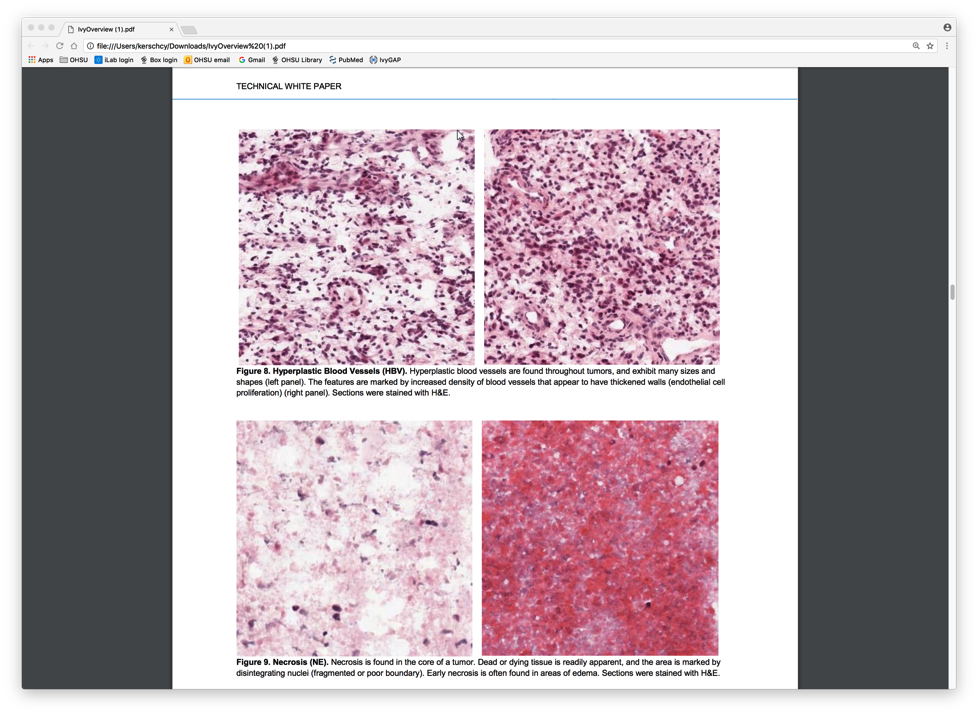Hyperplastic Blood Vessels (HBV) |  | “Hyperplastic blood vessels are found throughout tumors, and exhibit many sizes and shapes (left panel). The features are marked by increased density of blood vessels that appear to have thickened walls (endothelial cell proliferation) (right panel).” |
| Pseudo-palisading Cells around Necrosis (PAN) | 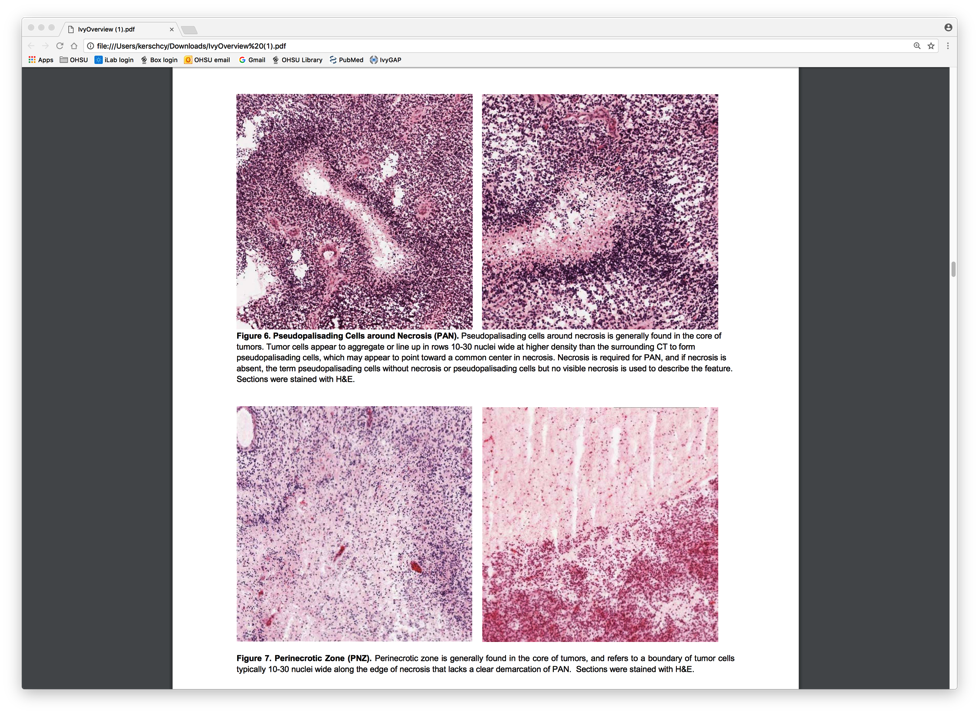 | “Pseudopalisading cells around necrosis is generally found in the core of tumors. Tumor cells aggregate or line up in rows 10-30 nuclei wide at higher density than the surrounding CT to form pseudopalisading cells, which may appear to point toward a common center in necrosis. Necrosis is required for PAN.” |
| Perinecrotic Zone (PNZ) | 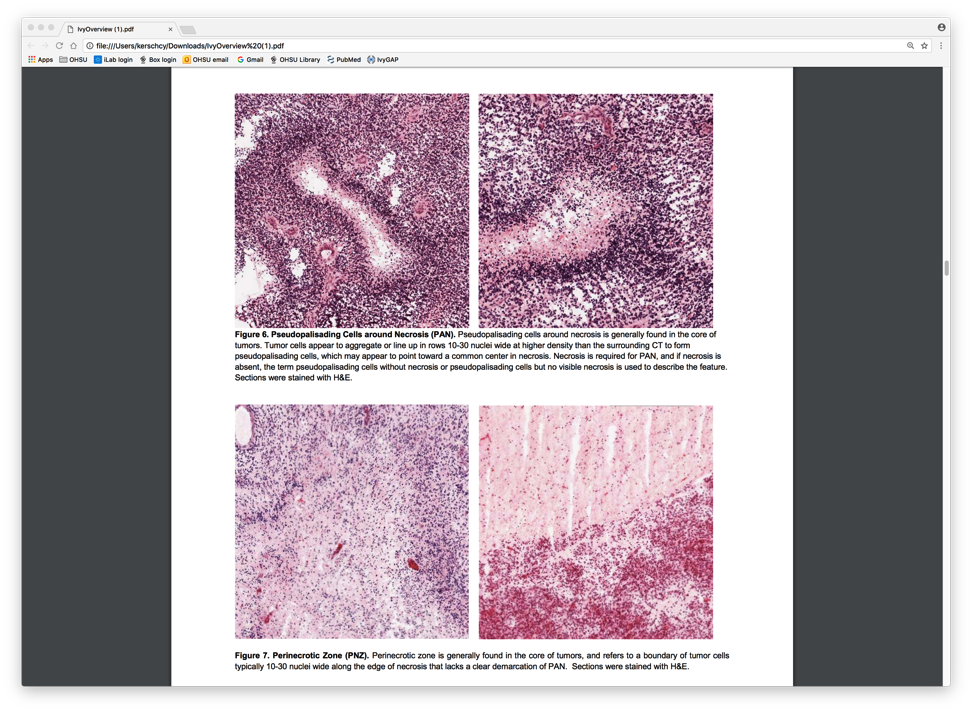 | “Perinecrotic zone is generally found in the core of tumors, and refers to a boundary of tumor cells typically 10-30 nuclei wide along the edge of necrosis that lacks a clear demarcation of PAN.”2 |
| Necrosis (NE)  **No RNAseq data for this structure* | 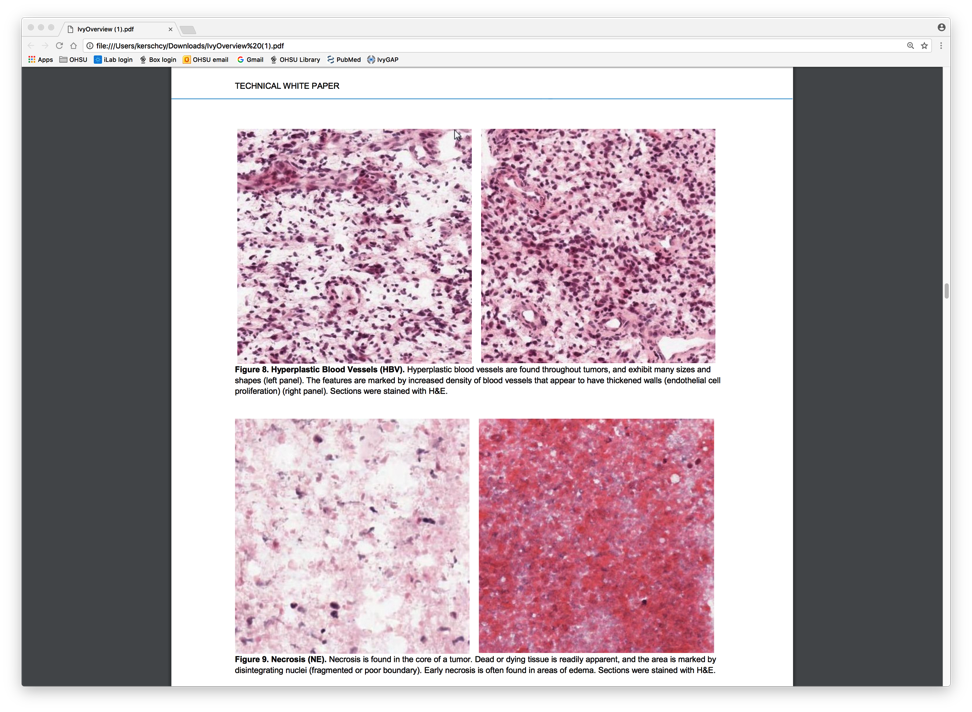 | “Necrosis is found in the core of a tumor. Dead or dying tissue is readily apparent, and the area is marked by disintegrating nuclei (fragmented or poor boundary).” |

**Table S2. New prognostic marker gene signature components.**


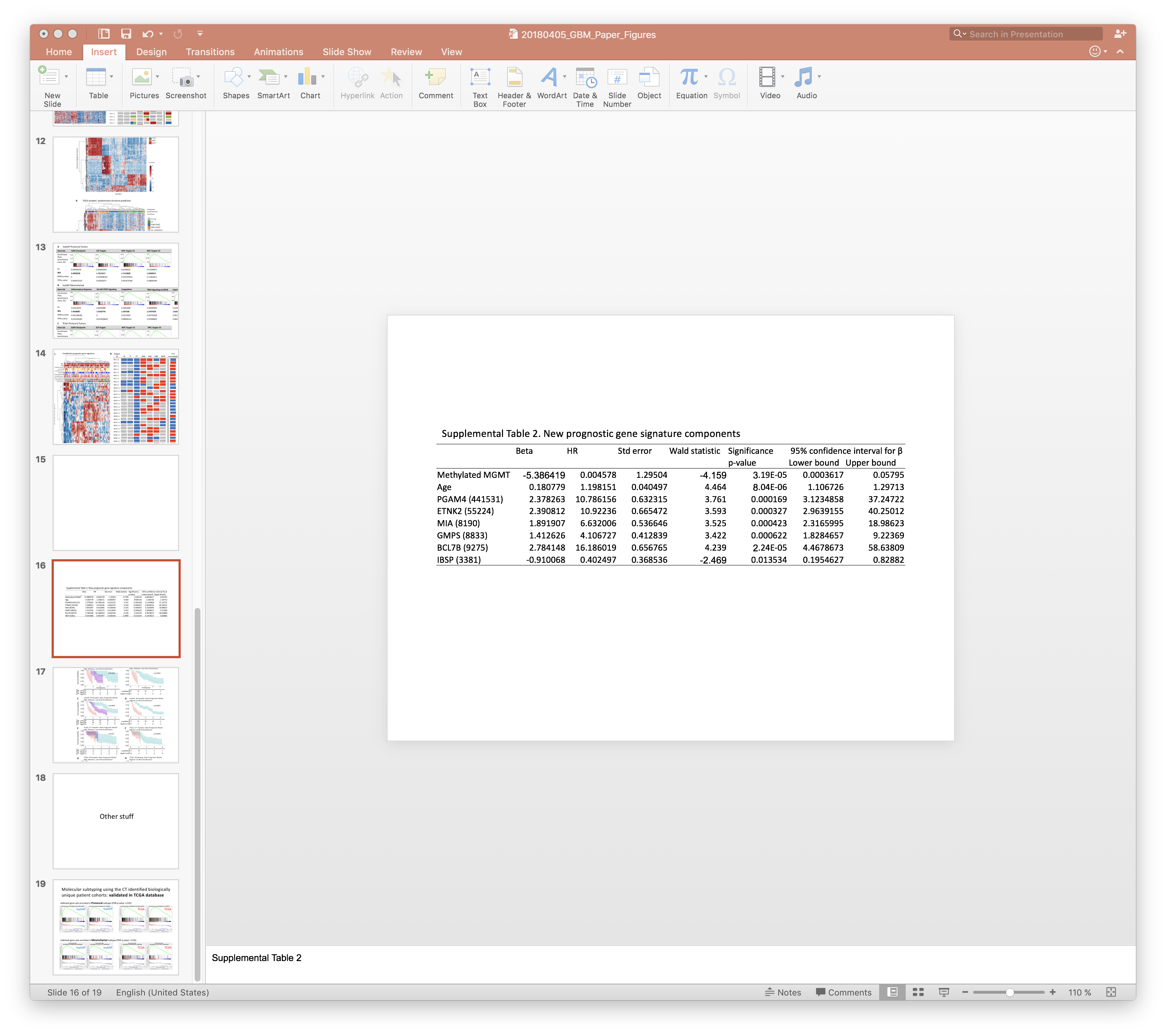


**Data files S1. Compiled code.** The following compiled text provides the code for data analyses completed in the submitted manuscript for reviewers. These files are uploaded to GitHub and will be made public and freely available upon acceptance of the paper for publication. The code compiled below includes: (1) Creating the IvyGAP heatmaps in R; (2) Creating the TCGA heatmaps in R; (3) Performing IvyGAP data transformations in R; (4) Performing TCGA data transformations in R; (5) Identifying the top 1000 most variable genes in R; (6) Performing PCA in R; (7) Collapsing structures in R; (8) Analysis of previously established prognostic gene set in R; (9) Coxph survival univariate and multivariate analysis in R; (10) Stepwise CV coxphanalysis in R; (11) Coxph survival model validation KM in R; (12) Logistic regression analysis in Python; (13) Balanced structures in Python.
